# PD-1/PD-L1 Checkpoint Inhibitors in Tumor Immunotherapy

**DOI:** 10.3389/fphar.2021.731798

**Published:** 2021-09-01

**Authors:** Jinhua Liu, Zichao Chen, Yaqun Li, Wenjie Zhao, JiBiao Wu, Zhen Zhang

**Affiliations:** ^1^ Innovation Research Institute of Traditional Chinese Medicine, Shandong University of Traditional Chinese Medicine, Jinan, China; ^2^ College of Pharmacy, Shandong University of Traditional Chinese Medicine, Jinan, China; ^3^ Experimental Center, Shandong University of Traditional Chinese Medicine, Jinan, China

**Keywords:** PD-1 inhibitor, PD-L1 inhibitor, biomarkers, immune checkpoint, tumor immune escape, immune-related toxicity, immunotherapy

## Abstract

Programmed death protein 1 (PD1) is a common immunosuppressive member on the surface of T cells and plays an imperative part in downregulating the immune system and advancing self-tolerance. Its ligand programmed cell death ligand 1 (PDL1) is overexpressed on the surface of malignant tumor cells, where it binds to PD1, inhibits the proliferation of PD1-positive cells, and participates in the immune evasion of tumors leading to treatment failure. The PD1/PDL1-based pathway is of great value in immunotherapy of cancer and has become an important immune checkpoint in recent years, so understanding the mechanism of PD1/PDL1 action is of great significance for combined immunotherapy and patient prognosis. The inhibitors of PD1/PDL1 have shown clinical efficacy in many tumors, for example, blockade of PD1 or PDL1 with specific antibodies enhances T cell responses and mediates antitumor activity. However, some patients are prone to develop drug resistance, resulting in poor treatment outcomes, which is rooted in the insensitivity of patients to targeted inhibitors. In this paper, we reviewed the mechanism and application of PD1/PDL1 checkpoint inhibitors in tumor immunotherapy. We hope that in the future, promising combination therapy regimens can be developed to allow immunotherapeutic tools to play an important role in tumor treatment. We also discuss the safety issues of immunotherapy and further reflect on the effectiveness of the treatment and the side effects it brings.

## Introduction

Through the process of tumor immune editing, tumor cells acquire multiple methods of evading host immunity in the tumor microenvironment (TME) (Dunn et al., 2002). Studies on tumor immune escape have shown that PD1/PDL1-mediated immune checkpoint in TME is an important component of the tumor immune escape mechanism (Inaguma et al., 2018; Prestipino and Zeiser, 2019; Zhang et al., 2020). Early preclinical evidence suggests that activation of PD1/PDL1 signaling pathway may be the mechanism by which tumors escape the antigen-specific T cell immune response (Dong et al., 2002; Iwai et al., 2002). PD1 on immune cells interacts with PDL1 on tumor cells can protect tumor cells from killing by immune cells (Carlomagno et al., 2017; Wang et al., 2018a; Takeuchi et al., 2020). PD1 was first described in the early 1990s as it is expressed in the course of inducing apoptosis in T cell hybridoma (Ishida et al., 1992; Agata et al., 1996). As research progressed, PD1 was found to be taken part in the negative regulation of apoptotic T cell-mediated immunological reaction through binding to PD-L1 (Nishimura et al., 1999; Greenwald et al., 2005). Studies have shown that immunotherapy is effective in treating melanoma and renal cell carcinoma, etc. (Siegel et al., 2017; Sanmamed et al., 2018; Yu et al., 2019). Recent years, checkpoint inhibitors targeting the PD1/PDL1 or Cytotoxic T lymphocyte associated protein 4 (CTLA-4) pathways have shown great success, and driven the development of immunotherapy (Lesokhin et al., 2015; Sharma et al., 2015; Shin and Ribas, 2015; Topalian et al., 2015; Søndergaard et al., 2018). The anti-CTLA-4 antibody ipilimumab has shown durable anti-tumor activity and prolonged survival in patients with advanced melanoma, but is prone to immune-related adverse events (IAEs) (Buchbinder et al., 2016). PD1/PDL1 inhibitors are promising immunotherapeutic agents that can achieve satisfactory efficacy for different tumor types, different treatment routes, different drug combinations and different treatment regimens (Chen et al., 2021). The incidence of PD1/PDL1 inhibitor-mediated IAEs was significantly lower compared to CTLA-4 blockade (Ott et al., 2013). Study shows that PD-1 pathway blockade is more efficient than CTLA-4 blockade in advanced melanoma (Farolfi et al., 2012).

## Biological Function of PD1/PDL1 in Tumor Immunity

PD1 is a checkpoint protein and a composition of the CD28 family. It pertains to a group of suppressor T-cell receptors that was not expressed by T cells alone, but was upregulated by antigen stimulation and cytokines caused by T cell excitation (Kinter et al., 2008; Kulpa et al., 2013). PD1 is also expressed by B cells, monocytes, and dendritic cells (DCs)( Keir et al., 2008), and regulates various aspects of its immune function (Thibult et al., 2013; Roy et al., 2015). PDL1 is a type 1 transmembrane glycoprotein of the B7 ligand family. Which is not only expressed on activated T cells and B cells but also on some non-hematopoietic cells (Zou et al., 2016). It is in a favorable position to regulate T cell function in DCs and other antigen-presenting cells (APCs). T cells recognize tumor cells in the human body and kill them, but when tumor cells recognize PD1 protein on T cells, the tumor cells will upregulate the PDL1 protein and PD1 binds to PDL1 leading to apoptosis of the T cells (Li et al., 2015; Topalian et al., 2016; Tremblay et al., 2018; Li et al., 2020).

**FIGURE 1:**
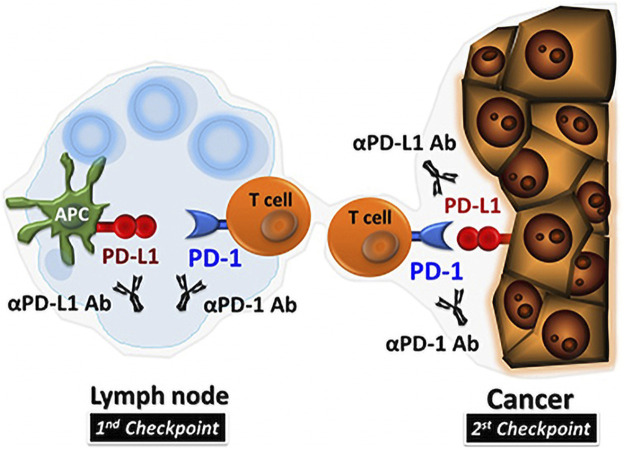
PD1/PDL1 inhibitors in TME (Hamanishi et al., 2016).

PDL1 on the surface of tumor cells can be upregulated by interferon gamma (IFN-γ) produced by activated T cells (Tang et al., 2018). PD1/PDL1 signal transduction pathway is a vital component of tumor immunosuppression, which can inhibit the excitation of T lymphocytes and strengthen the tumor cellular immune tolerance, so as to achieve tumor immune escape (Iwai et al., 2017). In summary, PD1 binds to PDL1 can diminish T cell-mediated immune surveillance, resulting in an absence of immunoreaction and even to apoptosis of T cells. It also inhibits tumor-infiltrating CD4+/CD8+ T cells (CD4+/CD8+ TILs) and leads to a decrease in cytokines including tumor necrosis factor (TNF), IFN-γ and Interleucina-2 (IL-2), so as to provide a way for cancer cells to escape the immunoreaction (Francisco et al., 2010; Daassi et al., 2020). PD1/PDL1 inhibiters unblock the immune suppression of anti-tumor T cells (Figure 1), which results in T cell multiplication and permeation into the TME and inducing an anti-tumor response (Kuzume et al., 2020). Existing anti-PD1/PDL1 therapy interdicts the combination between PD1 and PDL1, and effectively activates depleted immune cells and triggers an anti-tumor immune response (Ribas et al., 2018; Seidel et al., 2018; Liang et al., 2021).

## Mechanism of Action and Treatment of PD1/PDL1 Inhibitors

### Peptides/Polysaccharides and Small Molecules Target Treatment

Recently, a great number of research has been devoted to the exploitation of peptide-based inhibitors and nonpeptidic small-molecules targeting PD1/PDL1 (Akturk et al., 2018; Sasikumar et al., 2018). Furthermore, through structural modification of peptidomimetic inhibitors, small molecules can be developed. Compared to monoclonal antibodies, small-molecule drugs offer significant advantages (Zhang et al., 2020).

### Peptide-Based PD1/PDL1 Inhibitors

The first inhibitor AUNP-12, which was reportedly patented in 2014 (Sasikumar et al., 2019), is a 29-amino acid branching peptide. In an animal study, tumor cell growth and metastasis were effectively inhibited by AUNP-12 with few adverse reactions. In addition to AUNP-12, other peptide-based PD1/PDL1 inhibitors also have been developed. For example, a small peptide mimicking a peptide containing 7–8 amino acids, showed the best bioactivity in mice infected with melanoma B16F10 cancer cells, reducing lung metastases by 64 percent. Another compound is a cyclopeptide derivative of 7–9 amino acids, characterized by the formation of a circular structure by an amide bond between the N and C ends of the amino acid residues. In Crystal Field Stabilization Energies (CFSE) detection, Sasikumar et al. found that a cyclic peptide derivative can induce the proliferation of spleen cells in mice with high expression of PDL1 in human breast MDA-MB-231 cancer cells and reduce the lung metastasis of mice with melanoma B16F10 cancer cells by 54% (Sasikumar, 2013; Sasikumar et al., 2015).

### Nonpeptidic PD1/PDL1 Small-Molecule Inhibitors

The first reported small molecule inhibitor based on the PD1/PDL1 axis was a derivative of sulfamethoxine and sulfamethimazole antibiotics (Sharpe et al., 2018), which have low cytotoxicity. They can block the PD1 signaling pathway through restraining the combination of mPD1 to mPDL2 within the micromolar concentration range. Recent years, Bristol-Myers Squibb (BMS) has revealed a large number of non-peptide small molecule inhibitors targeting PD1/PDL1 pathway (Chupak et al., 2015). Among reported compounds, the representative compounds BMS-8 had IC50 value of 146 nM, and BMS-202 had IC50 value of 18 nM. Researchers investigated the mechanism of action of BMS inhibitors and demonstrated that these inhibitors induce the dimerization of PDL1, thereby suppressing the activation of PD1 (Zak et al., 2016). Holak’s team showed that some BMS compounds have structures that bind directly to PD-L1. More importantly, the combination between PD1 and PDL1 was blocked by inducing and stabilizing the formation of PD1/PDL1 homodimer under the action of compounds (Sasikumar et al., 2016). The IC50 values of representative compounds LH1306 and LH1307 were 25 and 3.0 nM, respectively. In addition, these inhibitors can interfere with interactions between PD1/PDL1 proteins and block PD1 signal transduction in co-culture experiments (Yang and Hu, 2019).

### Aptamer Therapy

Aptamer-Drug Conjugates (APDCs) are a very promising platform. Studies have shown that APDC can deliver immunomodulators, restrict immunomodulatory co-stimulation to tumor regions, induce neoantigens in tumors, block depletion-induced immune checkpoints, activate functional immune cells and prolong anti-tumor immunity (Zhu and Chen, 2018). Geng et al. designed and synthesized an amphiphilic telomeric dimer, aptamer polyvalent drug conjugate (ApMDC). And described the use of ApMDC nanoparticles to enhance the antitumor reaction of α-PD1 immunotherapy with targeted chemotherapy to tumors (Geng et al., 2021). They established 4t1 (breast cancer cell) and h22 (hepatoma carcinoma cell) tumor-bearing mouse models and draw a conclusion that the increased antitumor immunity accelerated the therapeutic reaction of α-PD1. In one study, researchers developed a DNA inducer for PD1/PDL1 signaling pathway to reverse immune evasion and stimulate antitumor immunity (Prodeus et al., 2015). DNA aptamer blocks the interaction of PD1/PDL1 by specifically binding to the extracellular domain of mouse PD1. MP7 is one of the aptamers, which can inhibit the inhibition of IL-2 secretion by primary T cells mediated by PD-L1. PEGylated MP7 directly blocks PD1 binding to PDL1. The Pegylated form of MP7 is equivalent to the antagonistic PD1 antibody, and can significantly inhibit the growth of PD-L1+ colon cancer cells *in vivo* for it retains the ability to block the PD1/PDL1 interaction (Ellington et al., 1990; Tuerk et al., 1990; Keefe et al., 2010).

**FIGURE 2:**
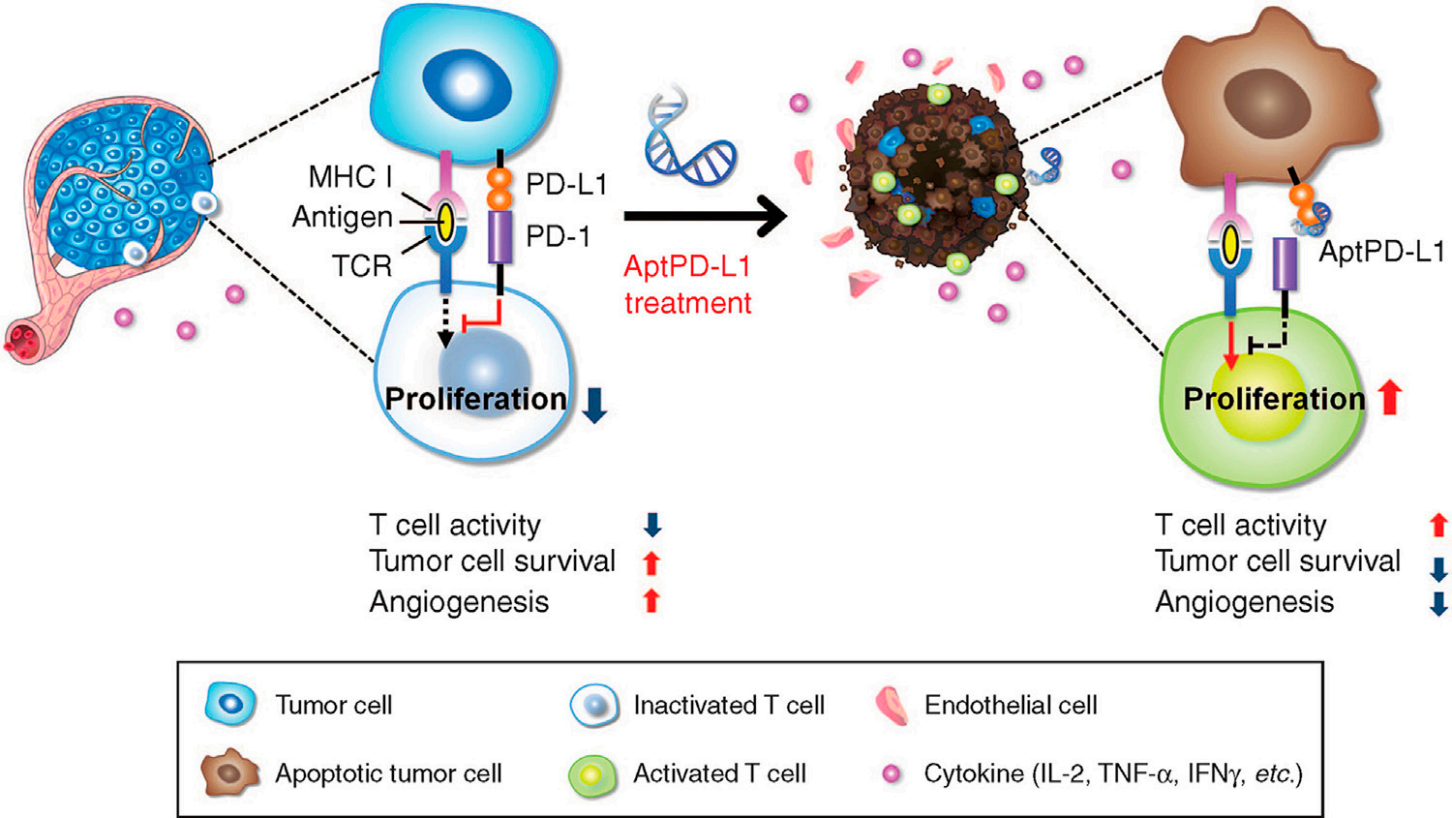
APTPD-L1 can inhibit the PD1/PDL1 interaction and weaken the inhibition of T cells (Lai et al., 2016).

According to another study, aptPDL1 stop the combination between PD1 and PDL1 in humans. Experiments in mouse models have shown that aptPDL1 promotes lymphocyte proliferation *in vitro* and inhibits tumor growth *in vivo* without causing significant hepatorenal toxicity. Further analysis of tumors treated with aptPD-L1 revealed increased levels of invasive CD4^+^ and CD8^+^ T cells, IL-2, TNF-α, and IFN-γ(Figure 2). Chemokine receptor 3(CXCR3) expression was higher in CD8^+^ T cells treated with aptPD-L1 than in tumors treated with random sequence oligonucleotide (Lai et al., 2016). Researchers have developed a novel PDL1 aptamer, a short single strand of DNA that is smaller than the PDL1 antibody, which can effectively avoid the effects of glycosylation that block PD-L1 binding. The selected adapter is more possibly to be glycosylated by PDL1 as peptide antigens, which is hopeful to provide a higher effectiveness of recognition while compared with PDL1 antibodies from extracellular cells (Huang et al., 2020). Liu’s team found that in the presence of dual targets (PDL1 as a natural receptor and azide modified glycoprotein as a chemical receptor), the cyclooctyne-coupled PDL1 (D-APDL1) can be covalently coupled to the surface membrane of cancer cells through APDL1 aptamer recognition and DNA logic calculation reaction of cyclooctyne/azide biological orthogonal reaction. This in turn triggers precise and sustained T cell-mediated anti-tumor immunotherapy (Yang et al., 2021). Besides, they also found that this logical calculation could achieve long-term retention in the tumor by inducing covalent coupling of the PDL1 aptamer on the tumor cell surface, thus providing effective and precise checkpoint-blocking immunotherapy.

### Antibody Therapy

Antibody-based inhibitors of PD1/PDL1 induce persistent tumor remission in various kinds of advanced cancer patients, making inhibition of the PD1/PDL1 signaling pathway clinically important in the treatment of tumors. So far, Food and Drug Administration (FDA) has approved six monoclonal antibodies targeting PD1 (nivolumab, pembrolizumab, and cemiplimab) or PDL1 (atezolizumab, Durvalumab and avelumab) for the treatment of hematological and solid malignancies. (Tan et al., 2016; Chen et al., 2021). Monoclonal antibodies (mAb), known as checkpoint inhibitors, overcome the shortcomings of traditional anticancer therapies and inhibit the PD1/PDL1 mutual effect. Using *in vivo* and *in vitro* studies, Lussier et al. have found that T cell function can be enhanced by blocking PD1 with antibodies (Lussier et al., 2015). Within tolerable limits, monoclonal antibodies can significantly reduce toxicity, reduce solid tumor size, inhibit advanced tumors and metastases, and improve overall survival in patients. Nivolumab and pembrolizumab have been given permission for the therapy of terminal melanoma, non-small cell lung cancer (NSCLC) and renal cell carcinoma (RCC) by targeting PD1 and blocking its interaction with PDL1 and PDL2 (Hughes et al., 2016; Arranz-Nicolás et al., 2021). Phase I clinical trials of pembrolizumab or atezolizumab in patients with mTNBC showed promising results, with objective response rates (ORR) of 18.5 and 33%, respectively (Hwang et al., 2019). However, due to its long half-life and binding time with the target, it is easy to result in severe immune-related adverse reactions. Besides, mAb drugs are expensive, complex to produce, and difficult to store and transport. Therefore, how to use the PD1/PDL1 signaling pathway to develop simple and efficient non-monoclonal antibody treatment strategy is the focus of our current work (Pan et al., 2021).

### Combination Therapy

In a study, the researchers used an immune rejection phenotype in a mouse model to discover that therapeutic application of TGF-β blocking antibodies in combination with anti-PDL1 reduces stromal TGF-β signaling, promotes T cell infiltration into tumor centers and stimulated powerful anti-tumor immunity ultimately leading to tumor extinction (Mariathasan et al., 2018). The addition of the anti-PD-L1 drug atezolizumab to NAB-paclitaxel chemotherapy has been shown to significantly improve PD-L1-positive (PD-L1+) metastases and improve overall survival (OS) in patients with advanced TNBC(Schmid et al., 2018). Combined use of CDK4/6 and PD-L1 inhibitors significantly increased the survival rate of mouse xenograft models (Zhang et al., 2015). It has been reported that combining PD1/PDL1 inhibitors with PARP inhibitors is hopeful to remarkably improve the overall efficacy of TNBC. Mechanistically, PARPI inactivates GSK3β, thereby enhancing PARPI-mediated upregulation of PDL1 and reducing the efficacy of PARPI through cancer-associated immunosuppression. Blocking PD-L1 can restore the weakened anti-tumor immune function and enhance the antitumor effect of PARPI.

When anti-PD1/PDL1 antibody (anti-PD1/PDL1) and anti-CTLA4 antibody (anti-CTLA4) are administered alone, their effectiveness is only 20–25% at most. When combined, the yield of anti-PD1/PDL1 and anti-CTLA4 could reach 60% (Wei et al., 2018). The combination of antibodies blocking PD1 and agonistic antibodies triggering the costimulatory receptor glucocorticoid induced tumor necrosis factor receptor (GITR) may further improve the therapeutic effect (Wang et al., 2018b). Using the mouse colon cancer cell line MC38, Wang et al. found that anti-PD-1+ anti GITR had a significantly stronger anti-tumor effect in mice than either antibody alone. The synergistic effect of anti-PD-1+ and anti-GITR depends on CD8^+^ T cells, which can directly kill cancer cells and are adept at recruiting other tumor oncogenic immune cells from tumor (Wei et al., 2018). Wang et al. used gemcitabine (GEM) and PD1/PDL1 checkpoint inhibitor to form reactive oxygen species reactive scaffold *in situ* for combination treatment. They found that aPDL1-GEM scaffold induced an immunogenic tumor phenotype in mice bearing tumor, promoted immune-mediated tumor regression, and prevented tumor recurrence after primary resection (Wang et al., 2018c). Gao et al. treated a mouse tumor model with an anti-PD1 antibody and an HDAC2 inhibitor. The combination of HDAC2 inhibitors and anti-PD1 antibodies obviously slowed tumor growth and improved survival compared to the anti-PD1 treatment group (Gao et al., 2020).

### Mechanism of Drug Resistance in PD1/PDL1 Inhibitor Therapy

Although immune checkpoint blocking therapy has achieved great success in clinic, the response rate of immunotherapy is still low (Spranger et al., 2015; O'Donnell et al., 2017). Research has suggested that only 10–30% of the patients can produce long-term and sustained efficacy after receiving PD1/PDL1 inhibitors. The majority of patients have no obvious response to the treatment or will remain resistant to it (Andrews et al., 2019). The development of PD1/PDL1 antibody resistance involves many tumor-related processes, including PD-L1 expression, tumor neoantigen expression and delivery, related cellular signaling pathways, tumor microenvironment, and epigenetic modifications. The lack of tumor antigens causes T cells to fail to recognize PD1/PDL1 antibodies, leading to drug resistance. In addition, molecules that process and deliver antigens, such as MHC class I molecules and β2 microglobulin, can also lead to resistance to immune checkpoint inhibitors (ICIs) when their genetic code is altered (D'Ursoet al., 1991; Restifo et al., 1996; Sucker et al., 2014). Aberrant cell signaling is also a factor contributing to immunotherapy resistance, such as the PI3K/Akt pathway, Wnt/β-linked protein pathway, JAK/STAT/IFN-γ pathway, and mitogen-activated protein kinase (MAPK) pathway (Munn and Mellor, 2013; Lin and Zhao, 2015).

### Immune-Related Adverse Events

Despite the promising efficacy of immune checkpoint inhibitors, the majority of treated patients have had immune-related adverse events (IAEs) to varying degrees (Reynolds et al., 2021). Commonly reported IAEs include rash or pruritus, gastrointestinal disorders, and endocrine disorders (Farolfi et al., 2012; Robert et al., 2015). Among these, cardiovascular toxicity is particularly severe. In recent years, reports of myocarditis associated with ICIs have increased (Moslehi et al., 2018). Myocarditis associated with ICIs often manifests as arrhythmias and can coexist with myocarditis and myasthenia gravis, with severe disease and poor prognosis (Hu et al., 2019). There is evidence that redox mechanisms are the main mechanism responsible for cardiotoxicity (Tocchetti et al., 2019). It has been shown that ICI treatment group had a higher incidence of cardiovascular adverse reactions than the non-ICI treatment group, and the incidence of cardiovascular adverse reactions was higher in patients treated with the combination of ICI + ICI than with ICI monotherapy. CTLA-4 is prone to immune-related adverse reactions such as rash, diarrhea, colitis, hepatotoxicity and endocrine disorders (Hodi et al., 2010; Gangadhar et al., 2014), as well as cardiotoxicity including pericarditis and myocarditis (Geisler et al., 2015; Heinzerling et al., 2016). As for PD1/PDL1 inhibitors, myocarditis has been reported after treatment with nivolumab (Honton et al., 2014) or pembrolizumab (Läubli et al., 2015). Wang et al. found significantly higher rates of colitis and diarrhea after receiving the combination of ipilimumab and PD1/PDL1 inhibitors than with a single agent (Wang et al., 2017).

## Conclusion and Prospect

Over the past 20 years since the discovery of PD1, numerous experimental studies have proved the clinical efficacy of PD1 blockers in a wide range of solid and hematologic malignancies, offering promising prospects for cancer patients (Errico, 2015). In addition, reports based on the clinical application of PD1 inhibitors have elucidated the mechanism of tumor immune escape and confirmed the general significance of tumor immune monitoring and tumor immune editing (Burnet, 1967; Schreiber et al., 2011). Nevertheless, there is still a need for a large number of basic and exploratory studies on the prediction of tumor biomarkers, as well as the efficacy of drug therapy and adverse drug reactions. However, this does not prevent PD1/PDL1 from being a key area of research. For the reason that PD1/PDL1 plays a crucial role in most cancers, the development of immunotherapy with blocking agents will undoubtedly be a huge opportunity and challenge. Due to the occurrence of drug resistance, the efficacy of immunosuppressive therapy is poor. We hope that future studies can minimize drug resistance, reduce the occurrence of immune-related adverse events and improve the efficacy of immunotherapy. We believe that as research progresses, personalized immunotherapy will be further developed in the clinic to bring hope to cancer patients.
